# On Diseases of the Liver

**Published:** 1846-08

**Authors:** 


### PART II.—REVIEW.

### ARTICLE V.

*On Diseases of the Liver.* By GEORGE BUDD, M.D., F.R.S., Professor of Medicine in King's College, London, and Fellow of Caius College, Cambridge. With colored plates and numerous woodcuts. Philadelphia: Lea & Blanchard. 1849. pp. 392. (From the Publishers.)

Diseases of the Liver are, doubtless, more common in the South and West than any other local affections, and it has been a great defect with our recently published bibliographical collections, and a source of regret and perplexity to our physicians, that whilst, of late, much valuable information has been disseminated with regard to the minute anatomical structure and healthy action of this organ, comparatively little has been written illustrative of its diseases, and pathological changes.

While modern authors have been very successful in availing themselves of the advantages afforded by important recent discoveries in anatomy and physiology, in treating of diseases of the nervous system, skin, kidneys, &c., the newly acquired information with regard to the structure and functions of the liver, seems not, till recently, to have served any very important purpose in enlightening us concerning its diseases.

It seems to us that Dr. Budd, in his work, the title of which we give above, has been most successful in his efforts to supply this deficiency, and, in our opinion, has furnished the profession with a monograph upon diseases of the liver, which, for perspicuity of style, sound scientific views, and methodical arrangement, is not surpassed by any of our best modern productions.

The most recent information concerning the structure and functions of the liver, not having been published in our text books, upon anatomy and physiology, in most common use, it is reasonable to suppose that many practitioners have not, as yet, had the means of acquiring this most important preliminary information. Dr. Budd, therefore, very wisely, as we think, previously to treating of individual diseases of this organ, gives us an introduction upon the "structure of the liver, upon the causes of the variations in its form, size, and color, and upon the physical qualities and composition of the bile, &c."

The structure of the Liver may be described very briefly, as being composed of hepatic arteries, hepatic veins, portal veins, biliary ducts, with their respective capillary vessels, duly supplied with absorbants, all united together by a dense fibrous membrane.

Like all other secreting mucus membranes, that, lining the secreting tubes and the hepatic ducts, in which they terminate, is covered by a layer of epithelial cells, by which the biliary matter is eliminated or separated from the blood circulating in the portal capillaries.

As to the distribution of blood vessels in the liver, it may be remarked that the portal vein enters the concave surface of the organ, and, after dividing and subdividing into numerous branches, breaks up at last, into innumerable very minute capillary vessels, to form an intricate network around the secreting epithelial cells, the blood not employed by these cells, to form bile, passes into other minute vessels,—the capillaries of the hepatic veins,—and from thence to the right side of the heart, to mingle with the systemic blood.

It appears then, that the capillaries of the portal vein, terminate in those of the hepatic, and that this net work of minute vessels, serves as the medium of connection between them.

This vascular plexus, situated between the termination of the portal and commencement of the hepatic vein, is separated into lobular masses by the larger blood vessels and hepatic ducts. These lobules, thus formed, together with the biliary ducts, and their secreting epithelial cells, constitute the so called, lobules of the liver.

Each of these lobules has its exterior composed, principally, of biliary ducts and branches of the portal plexus, having a small hepatic vein with its capillaries originating in its centre. This arrangement of vessels enables us by the appearance of the lobules, to distinguish between congestion of the hepatic and portal veins, and to detect, also, the undue accumulation of bile in the hepatic ducts.

In diseases of the liver, caused by obstruction to the passage of blood through the hepatic veins, the lobules present a dark vascular centre, with a yellowish or light colored circumference; an appearance produced by an accumulation of blood in the capillaries of the hepatic vein, and a comparatively empty condition of the portal plexus and minute biliary ducts. On the other hand, in congestion of the portal veins and their capillaries, the dark vascular rings, surrounding light colored centres, presented by the lobules, indicate, not an obstruction to the passage of blood through the hepatic veins, but an accumulation of it, in the vena potarum and its branches. Obstructions to the passage of bile from the liver, and a consequent accumulation of it, in the hepatic ducts, cause the whole organ, but especially the exterior of the lobules, where these minute ducts are most numerous, to present a dark yellow appearance. Thus it is seen that obstruction to the passage of fluid, may take place in either of the above named three classes of vessels, causing hepatic venous, hepatic portal, or hepatic biliary congestion, each depending upon its own cause, and producing its peculiar pathological changes and appearances.

The hepatic artery, upon entering the liver, divides into numerous branches, which are distributed, largely, to the coats of the blood vessels, and in great numbers to the hepatic ducts, gall bladder, cystic, and common ducts. The arterial blood, thus distribnted, is taken up by small branches of the portal vein, and, like that from the gastric, splenic, superior and inferior mesenteric arteries, passes in the liver, through a second set of capillaries, the portal plexus.

It is the opinion of most physiologists, founded, partly, upon this anatomical distribution of vessels, that the arterial blood of the hepatic artery serves as nutriment for the liver, and that the venous blood of the portal vein, furnishes the material for its secretion.

Having given the above brief description of the distribution of the vessels of the liver, we will now pass to the consideration of some of the other elements of its structure.

As before remarked, the meshes of the portal plexus or spaces between the vessels which constitute the lobules, are filled up with numerous nucleated secreting cells. The language of our author upon this subject is not susceptible of abridgment and may therefore be quoted. Concerning the structure and functions of these cells, he remarks as follows:

"The cells are flattened, irregular in form, but somewhat spheroidal, and have each a nucleus, which again contains a central pellucid spot, the nucleolus. Some cells have two nuclei.

"The cells are of various sizes. The largest are usually about the one-thousandth of an inch in diameter. Others are very much smaller, as if not yet fully developed. In some livers the cells, generally, are smaller than in others.

"The cells contain oil-globules and amorphous granular matter. Their colour and transparency depend on the colour and quantity of the matter they contain, which vary very much in different cases. They are usually of a light brown and almost transparent, but in some subjects we find them yellowish and opaque.

"If, while looking at this mass of nucleated cells, we imagine the delicate and now invisible capillaries to be filled with blood, or coloured size, and thus rendered conspicuous, we shall perceive that the whole liver, excluding the canals in which the portal and hepatic veins run, is a solid plexus of capillary blood-vessels, the meshes of which are filled with nucleated cells.

"The mucous membrane of the gall-bladder and ducts, like the excreting ducts of other glands, in fact, like all mucous membranes, and the skin itself, is composed, as Mr. Bowman has shown, of an extremely thin, transparent membrane, without pores or visible structure, whose external or secreting surface is coated with nucleated cells. These cells by their apposition and union, form a kind of pavement on the transparent membrane, which serving as their basis of support, has for this reason been named by Mr. Bowman, the *basement-membrane*. The blood-vessels, lymphatics, and nerves ramify on the opposite, deep, or inner surface of the basement-membrane."

"The researches of Purkinje, Henle, Bowman, and Goodsir, leave no doubt that the nucleated cells are the immediate agents of secretion.

"It is not in the liver only that the cells perform this office, for it seems established as a general law, and it is certainly one of the highest and most interesting which the study of minute structure has yet disclosed—that all true secretion, whether in animals or in plants, is effected by the agency of cells; that, 'however complex the structure of the secreting organ, these nucleated cells are its really operative part.' In each secreting organ, the secreting cells have a peculiar power to form, or to withdraw from the blood, the secretion proper to the part.

"In such of the glands of animals as have excreting ducts, the nucleated cells withdraw from the blood the peculiar principles of the secretions, which they elaborate more or less, and then, in one way or another, whether by bursting or dissolving, or by some unknown mode, discharge them through the excreting ducts.

"The evidence of this is, perhaps, as clear in the liver as in any of the glands.

"On examining the neucleated cells of the liver under the microscope, we see that most of them enclose small spheroidal globules, which are recognized by their dark outline, or high refractive power, to be globules of oil or fat.

"In ordinary livers these oil or fat globules are small, and few in number; but in the fatty condition of the liver, so often found in persons dead of phthisis, and in that induced by keeping animals exclusively on fatty substances, they are so large and numerous as to distend the cells to double their natural size, and consequently to cause a great increase in the volume of the liver."

Having described the most important parts which constitute the liver, it remains to say a few words concerning its areolar tissue and lymphatic vessels.

The areolar tissue seems to connect together and protect the more important elements of the organ, and in man, is spread out over its whole surface, and by forming tubes, called portal canals, surrounding the ducts and portal veins, extends into its interior, constituting the medium of connection between its different parts. This structure is interesting in a pathological point of view, as will be seen hereafter, on account of its being the part primarily and principally effected in cirrhosis.

As for the lymphatics, those which are superficial, ramify in the proper capsule of the liver, and the deep seated ones, in the portal canals.

"Having examined the structure of the liver, we may next consider the composition and uses of the bile.

"We have seen that the neucleated cells in the lobules of the liver withdraw from the blood the principles of their secretion, which they probably elaborate in some degree, and then discharge into the ducts. In its passage through the ducts, the matter secreted by the lobules becomes mixed with that secreted by the ducts themselves, which, if we may judge from the large quantity of blood the ducts derive from the hepatic artery, and the numerous involutions of their mucous membrane, must be considerable in quantity. Secretion is always going on, both in the lobules and in the ducts, and the compound fluid derived from these two sources probably passes continuously along the ducts as far as the junction of the hepatic duct with the cystic.

"When the stomach and duodenum are empty, part only of the bile flows along the common duct into the duodenum; the remainder passes down the cystic duct into the gall-bladder.

"During digestion, on the contrary, the gall-bladder contracts, and part of the bile accumulated in it, together with all that brought by the hepatic duct, is poured into the duodenum.

"In the gall-bladder, the bile loses, by absorption, some of its most watery parts, and is further modified by the addition of the proper secretion of this cavity."

"The bile, in man, has been supposed to be ultimately derived from two sources. It is clear enough that, in most circumstances, a large proportion of the proper principles of bile are derived from the waste of the body, and are a product of the metamorphosis of the tissues and of materials stored away in the system. In the carnivora, in the hybernating animal in its winter sleep, and in the fœtus, these materials must be its only source. And under certain conditions, the same must be the case in man also. In protracted abstinence, for example, bile continues to be formed, and often in large quantities. Here, the living tissues gradually waste away, and their materials are discharged in the excretions. The three principal outlets at which they make their appearance, are the liver, the lungs, and the kidney. Nitrogen predominates in the compounds which escape through the last-named organ, while the two former separate principally hydrogen and carbon. But while the liver and lungs have thus much in common, there is this important difference between them; that in the lungs, the hydrogen and carbon pass off *burnt*—that is, in combination with oxygen, as water and carbonic acid,—while, in the liver, they escape uncombined with oxygen, and still combustible. From which it would appear, that the larger the amount of these elements discharged by the lungs as water and carbonic acid, the less, *cæteris paribus*, must remain *unburnt* to form constituents of bile. So that here, we already meet with a fundamental and important relation between the secretion of bile and the great function of respiration. I shall not, however, dilate upon this topic now, as in endeavouring to follow the bile to its final destination, we shall again have to consider relations of a similar kind."

It is also probable, that in man, and in all animals which live on a mixed diet consisting largely of combustible matters, such as starch, sugar, alcohol, and other compounds destitute of nitrogen, these substances, also, contribute to the elements of the bile.

With regard to the part the liver performs in the animal body in secreting bile, it may be remarked, that physiologists, though much better informed than formerly upon this subject, are still unsettled in their opinions. One of the principal effects of the action of the liver in secreting bile is, evidently, to purify the blood, by separating from it noxious and effete principles.

"It will be remembered that all the blood sent to the stomach and intestines, has to pass through this organ before it can again mix with the venous blood from other parts of the body. Now the blood that has come from the stomach and intestines must necessarily be charged with many impurities besides those derived from the mere decay of the tissues. Along the extensive mucous tract, with which every thing we eat or drink is brought in contact, absorption is constantly going on, and various matters must, therefore, enter the portal vessels, not fit by their nature to form blood, or to serve any other purpose in the body. Many of these substances are removed from the blood in its passage through the liver. The discharge of such matters through the liver, when they are in unusual quantity, or of a particular kind, is, no doubt, the primary condition of many biliary disorders.

"But the bile is far from being a merely excrementitious fluid. Arrived in the intestine, it has important offices to serve, as indeed might already be surmised from its being poured into this canal so near its upper end. These offices are related to the function of digestion on the one hand, and (according to Liebig) to that of respiration on the other."

The bile is supposed to assist in digestion, by uniting by means of its alkaline constituents, with oils and fats, forming with them a kind of soap which is soluble, and also by means of the same principle, to neutralize the acid which passes from the stomach, after having performed its part in the process of digestion.

Another object of the bile is, doubtless, to promote the discharge of the contents of the intestines, by acting as a natural purgative. This is evident, from the fact, that an excess of bile is attended by diarrhea, and a deficiency by constirpation.

"We have next to consider the final destination of the bile itself. It seems clear that, in man, under ordinary circumstances, the bile which is evacuated by the bowel, can be but a small proportion of the whole amount secreted. For the quantity thus voided is very trifling, and consists chiefly of its colouring matter. The remainder, and larger part, must, therefore, be re-absorbed. Liebig states, that, in the carnivora, the whole of the bile is re-absorbed. The excrements of these animals contain neither bile nor soda; for water extracts from them no trace of any substance resembling bile, and yet bile is very soluble in water, and mixes with it in every proportion. It has been lately advanced by Liebig, on the authority of quantitative analysis, that the portion of bile re-absorbed, is eventually discharged through the lungs as carbonic acid and water; thus supplying fuel for respiration and supporting animal heat."

"Thus it appears, that the relation of bile to respiration is direct and fundamental. Fortunately, the activity and effects of the respiratory process are largely under our control. In the vast power we have of modifying these by appropriate regulations, having reference to the great conditions of air, exercise, temperature and food, we have means much more effectual than any other, in dealing with biliary disorders.

"Of these disorders, on the other hand, the neglect of such regulations is by far the most fruitful source.

"Thus, for example, may be explained many of the bilious disorders of hot climates. If, in such climates the food be not regulated in accordance with the smaller needs of the economy as to animal heat, an excess of bile is formed, and disorder of the stomach and intestines—bilious vomiting, and diarrhœa—are the consequence.

"Hence, also, the general repugnance to rich meats, and the greater tendency which these and spirits unquestionably have to produce disease of the liver, in hot seasons and in tropical climates.

"In the same way may be explained the greater frequency of bilious disorders in middle life, when men begin to take less exercise, and their respiration becomes less active, while on the other hand, the tendency to indulge at table but too often increases."

In the preceding remarks we have, as far as space will permit, given an exposition of the facts and principles laid down in our author's introduction. We shall now proceed to give a brief synopsis of his views and opinions with regard to individual diseases, as expressed in the following chapters.

Congestion of the liver is the morbid condition of the organ first treated of, and is described as being very common, from the fact, that it is composed mostly of vessels, and has circulating through it, a large proportion of blood, the portal, already retarded by its passage through one set of capillary vessels.

Congestion may take place in the hepatic or portal veins, or in the hepatic ducts. When from organic disease of the heart, or acute disease of the lungs, the course of the blood through the chest is impeded, there is, always, congestion of the hepatic veins.

The liver in such cases presents different appearances, according to the degree of congestion.

"In slight degrees, the twigs of the hepatic vein and the capillaries that terminate in them, are found, after death, turgid with blood, while the portal twigs, and the capillaries that immediately spring from them, are empty. A section of the liver presents, in consequence, a mottled appearance. The central portions of the lobules, where the vessels are congested, form isolated red spots, while the margins of the lobules, where the vessels are empty, have a colour which varies from yellowish-white to greenish, according to the quantity of oil-globules and of colouring matter which the cells contain. This appearance has been termed by Mr. Kiernan, the first stage of *hepatic-venous* congestion. When the course of the blood through the heart or lungs is impeded, the hepatic veins and the capillaries that open into them are naturally the first to become turgid.

"In a further degree of congestion, more of the vessels forming the capillary network are filled of course in a direction backward, towards the portal vessels. The congestion extends from lobule to lobule, at those points where the adjacent lobules are connected by their capillaries; and when the congestion has nearly, but not quite, reached those twigs of the portal vein that go to define the lobules, all the capillaries of the lobules will be injected, excepting those immediately surrounding the portal twigs. A section of the liver will still present a mottled appearance, but now the pale portion will be in spots, where the uninjected twigs of the portal vein are divided, and the red portion will form a band continuous throughout the liver. This appearance is what Mr. Kiernan has called the second stage of hepatic-venous congestion.

"A liver congested to this degree is enlarged from the large quantity of blood it contains; and, as Mr. Kiernan has remarked, it is frequently at the same time in a state of *biliary* congestion. The biliary congestion is an accumulation of biliary matter in the lobules of the liver, giving the uninjected portions of the lobules a deeper yellow or greenish tint than is natural to them. It seems to be a consequence of the congestion of the blood, and is produced perhaps in a great measure by impediment to the free escape of the bile through the small ducts, from the pressure exerted on them by the distended vessels."

"'When obstruction takes place to the circulation through the chest, but more particularly when the heart becomes over-distended with blood, we observe the countenance gradually assume a dingy aspect, in which the purple suffusion of carbonized blood is mingled with the yellow tint of slight jaundice: the conjunctiva is more decidedly tinged: and if the disease continue long, sometimes completely prevails over the purple tint.'

"This jaundiced tint of the complexion, co-exists with a jaundiced condition of the liver itself, or, as Mr. Kiernan expresses it, with *biliary congestion* which has already been noticed as sometimes consequent on sanguineous congestion.

"If the biliary congestion be long kept up, the function of the cells in the congested lobules is arrested, or rendered less active, and the cells become perhaps impaired in their vitality and powers of reproduction. The liver is permanently injured in its secreting element, as it is when the common duct has been long obstructed.

"Andral and most other writers have remarked that congestion of the liver from a mechanical cause, when long continued often leads to organic disease of the liver; and they have explained in this way the frequent association of organic disease of the liver with organic disease of the heart."

"There is little to be said on the treatment of mechanical congestion of the liver. The congestion is the consequence of another disease, and the treatment which relieves the latter, will diminish the congestion. When the congestion depends on obstacle to the circulation through the heart, the proper remedies are those,—such as bleeding, purgatives, diuretics, rest,—which most effectually relieve the heart. When the liver cannot free the blood from the principles of bile, or the skin becomes sallow, the patient should carefully abstain from rich meats and fermented drinks, which would render the liver still more inadequate to its office, and increase the bilious disorder."

"In congestion of the liver from disease of the heart and lungs, the hepatic veins, being nearer the seat of obstruction, in the course of the circulation, than the portal veins, are naturally the vessels first distended;—and when the congestion is partial, the hepatic twigs, and the capillaries that immediately surround them, are found after death, to be the full vessels; the portal twigs, and the capillaries that immediately spring from them, the empty ones.

"But now and then, the portal veins, and the capillaries immediately springing from them, are found alone congested. The margins of the lobules, and the interlobular spaces are then of a red colour—forming a continuous red band—while the centres of the lobules appear as isolated pale spots.

"Mr. Kiernan has applied to this congestion of the portal veins only, the term, *portal-venous* congestion. From the pale uninjected portion being in isolated spots, it looks very like the second stage of hepatic-venous congestion. It is remarked by Mr. Kiernan, that the injected substance never has the deep red colour that it has in hepatic-venous congestion."

Inflammatory diseases of the liver are next treated of by the author.

The old division of this class of diseases into acute and chronic is, in his opinion, faulty, from the fact, that those attacking the exterior of the organ, being productive of more tenderness and pain, are termed acute; whilst others, no less rapid in their course being deeply seated and consequently less tender and painful, are termed chronic.

The classification of inflammatory diseases is as follows.

1. Suppurative inflammation or that which leads to suppuration and abscess.

2. Gangrenous inflammation.

3. Adhesive inflammation, or inflammation which causes effusion of coagulated lymph.

4. Inflammation of the veins of the liver.

5. Inflammation of the gall bladder and ducts.

The first mentioned or suppurative inflammation and abscess of the liver may be caused, either by blows or other mechanical injury, by suppurative inflammation of veins, or by ulceration of parts from which blood returns through the liver.

Inflammation and abscess of the liver from external violence is so rare, and the treatment in such cases so obvious, that we will remark, merely, that its usual seat is upon the convex surface of the right lobe, and that the rarity of inflammation and abscess from accidental injury, shows how effectually the liver, when of its natural size, is shielded by the ribs.

"A second, and far more frequent cause of abscess of the liver, is suppurative inflammation of some vein, and the consequent contamination of the blood by pus."

"The mode of formation of these abscesses is well illustrated by an experiment made more than half a century ago by Dr. Saunders, and related by him in his admirable work on the structure and diseases of the liver. He injected ℨij of quicksilver into the crural vein of a dog. No ill effects were observed the first day, but at the end of this the dog became feverish, and after two or three days had cough and difficulty of breathing, which continued until its death. On examination after death, Dr. Saunders found the lungs studded with small indurated masses, which he calls tubercles, and small ciscumscribed abscesses. In the centre of each was a small globule of mercury.

"Here, the globules of mercury, like the globules of pus in purulent phlebitis, became arrested in the capillary vessels of the lungs, and each globule, acting perhaps by mere mechanical irritation, excited circumscribed inflammation and abscess. The inflammation was circumscribed, because the irritation that excited it, acted only at particular points.

"In the dog experimented on by Dr. Saunders, the lungs were the only organs in which abscesses were found. The reason of this is obvious. All the mercury, conveyed directly to the lungs, became arrested in their capillaries. No globules passed through to cause inflammation and abscess of other organs.

"In the same way, in some cases of purulent phlebitis consequent on injury of the head and limbs, or on amputation, abscesses are found in the lungs only; and they are usually found in the lungs in greater number than in other internal organs. After the lungs, the liver is the organ in which they are most frequent; a circumstance attributable, in some measure, to the large quantity of blood that flows to the liver, and to the slowness of the current through its capillary network; but, perhaps, still more, to those vital or other attractions by which matters of particular composition are there detained and excreted."

"There is a close analogy between the secondary abscesses from phlebitis, and secondary masses of cancer.

"A cancer of the breast may be the source of cancerous tumours in the lungs and liver, just as an inflamed vein in the arm may be the source of abscesses in those parts.

"The abscesses and the secondary cancerous tumours will be scattered in the same manner, and immediately surrounded by healthy pulmonary or hepatic tissue.

"The lungs and the liver are the organs in which secondary cancerous tumours, as well as the abscesses from phlebitis, are most frequent.

"The cancerous tumours and the abscesses have in each organ the same form and seat; and in the lungs, both have a great predilection for the surface.

"These points of resemblance can hardly be explained, except on the supposition that the germs of the two diseases, —cancer-cells and pus-glonules,—are disseminated in the same manner through the veins."

"The veins that feed the vena portæ, are little exposed to accidental injury, but some of their branches are divided in operations on the rectum and for strangulated hernia; and, as might have been anticipated, these operations are sometimes followed by abscess of the liver.

"Cruveilhier relates a case where abscesses of the liver were immediately consequent on repeated attempts to return a prolapsed rectum.

"The patient, a man of sixty, had been subject to prolapsus many years. The bowel protruded at the first effort to empty it, but was usually returned without difficulty. When he sought assistance on the last occasion, it had been down twenty-four hours, and was replaced only after repeated and violent attempts, which gave him much pain.

"The same day the expression of his countenance altered, and his pulse became small and unequal. He soon fell into a state of prostration, with a cold skin, vomiting, hiccough, stupor, but without pain, and died on the fifth day.

"A great number of small abscesses, some superficial, others deep-seated, were found in the liver. The hepatic tissue for a short distance round each of them was of a brown slate colour and softened. (Cruv. liv. xvi.)

"Dance mentions a case in which abscesses formed rapidly in the liver after an operation for cancer of the rectum, where cauterization was practised; another, in which they were consequent on a simple operation for fistula; two others, in which they follow the operation for strangulated hernia, where a portion of irreducible omentum suppurated externally. (Archiv. Generales, t. xix. p. 172.)

"There can be little doubt that in all these cases, the abscesses in the liver were the consequence of phlebitis caused by the operations."

The remaining causes of suppurating inflammation and abscess of the liver, are, as stated above, "ulceration of the large intestine, or more generally, of the intestines, the stomach, the gall-bladder or ducts; parts which return their blood to the portal vein, to be thence transmitted through the capillaries of the liver."

One of the diseases most frequently attended with ulceration of the intestines is dysentery, and it appears from numerous cases cited by our author, that whenever, in this disease, there is ulceration of the intestinal mucus membrane, abscess of the liver is also found.

Upon this subject he remarks:

"A connection between abscesses of the liver and dysentery has long been noticed, but the two diseases are associated far more frequently than has been generally imagined. Of the twenty-nine cases recorded by Annesley, there are twenty-one, or nearly three-fourths, in which there were ulcers, more or less extensive, in the large intestine; and two other cases, in which the large intestine was contracted or strictured, in consequence, no doubt, of dysentery at some former period. It is not unlikely that in some of the remaining cases, ulceration of the intestines existed but was not noticed.

"Of the fifteen fatal cases which fell under my own observation at the Dreadnought, the state of the intestines was not noticed in two. In eight of the remaining thirteen cases, there were ulcers in the large intestines, and in one other case, two ulcers in the stomach; so that, in nine of thirteen cases, or in nearly three-fourths, there were ulcers in the large intestine or stomach. In another of these cases, without ulceration of the stomach or intestine, there was ulceration of the common gall-duct."

After recording numerous other cases of dysentery attended by abscess of the liver, our author remarks, that "the association of dysentery with abscess of the liver, is noticed by most physicians who have treated of either of these diseases," and that "it is impossible to suppose that this is a mere coincidence of disease having no relation to each other."

Upon the manner in which the ulcerated mucus surface acts to produce hepatic abscess, his language is as follows:

"Admitting dysentery, or ulceration of the bowel, to be a source of abscess of the liver, it is obvious that the liver does not become involved by spreading of the inflammation, but by some contamination of the portal blood.

"This may be either by *pus*, formed by suppurative inflammation of one of the small intestinal veins; or by matter of other kind resulting from softening of the tissues; or by the fetid gaseous and liquid contents of the large intestine in dysentery, which must be absorbed and conveyed immediately to the liver. It seems probable, that contamination of the first kind usually gives rise to small scattered abscesses; of the last, to diffuse inflammation, and a larger, perhaps single, collection of pus. If the morbid matter be such, that it does not mix readily with the blood—as globules of pus or mercury—it will cause small, circumscribed abscesses, the rest of the liver being healthy. If, on the contrary, the morbid matter be readily diffusible in the blood, all the blood will be vitiated, and diffuse inflammation result.

"The admission of this explanation of the relation of abscess of the liver to dysentery, would lead us to expect that abscess of the liver might occasionally be consequent on ulceration of the stomach, or gall-bladder,—parts, which, like the larger intestine, return their blood to the portal vein,—and this is found to be the case."

As to symptoms it is stated that they are generally much more obscure than they are represented to be in books upon this subject. "A picturesque group is sketched, which it seems very easy to identify, but in actual practice it is far otherwise."

"We can only infer that abscesses are forming in the liver by the occurrence of *special* symptoms—pain in the region of the liver and jaundice—in the midst of the general disorder. But these special symptoms do not exist in all cases. There may be no jaundice; and pain, even, may be wanting, or the typhoid state into which the patient falls may prevent his distinctly perceiving or expressing it. In such cases, the abscesses in the liver can be discovered only after the death of the patient.

"In the same way, when inflammation of the liver occurs during the acute stage of dysentery, or on a recurrence of acute symptoms in chronic dysentery, the general symptoms do not aid us in discovering it, because they are fairly attributable to the primary disease. The diagnosis must be founded on local symptoms chiefly—pain and tenderness referrable to the liver, tension in the right hypochondrium, and jaundice. Our knowledge of the connection between the two diseases enables us to attach due importance to these symptoms, and ascribe them to their actual cause. Pain and tenderness in the region of the liver, slight increase in its volume, and jaundice, which, in other circumstances, might excite little alarm, and be attributed to their most frequent cause,—inflammation and abstruction of the gall-ducts,—when they occur in the course of dysentery, will lead us to dread suppurative inflammation and abscess."

"The *treatment* of suppurative inflammation of the substance of the liver is very unsatisfactory.

"When the inflammation is caused by phlebitis consequent on a wound or injury of the head or limbs, the whole mass of venous blood is contaminated by pus, suppurative inflammation is likewise set up in many lobules of the lungs, perhaps in some of the joints, and, it may be, in various other parts of the body; and the patient soon falls into a typhoid state, which bleeding and other lowering measures would only make worse. The inflammation thus excited passes rapidly on to suppuration, and we have little, if any, power to arrest it.

"The chief objects of treatment should be, to prevent, where this is possible, the passage of any more pus into the blood from the injured part, and to support the strength of the patient.

"When suppurative inflammation of the liver is caused by a blow, the lungs and other organs do not suffer as in purulent infection of the blood: neither are they thus implicated, when it is induced by ulceration of the stomach, or intestines, or gall-bladder, since, in these cases, the noxious matter which excites the inflammation, is detained in the liver or drained off through it. Here, the strength of the patient is not so profoundly sunk, and we may hope by means of depletion, especially local bleeding, to control the inflammation, and limit its extent; and, by rendering the abscesses smaller, to protract, at least, the patient's life. In some cases we may, perhaps, by active measures employed early, prevent matter from forming, but we have no evidence that this can be done when the inflammation is caused by pus, and is the consequence of inflammation of one of the veins that return their blood to the portal vein.

"In this country, mercury has generally been resorted to, when the local symptoms have led to the suspicion that the liver was diseased; but I fear, with no benefit to the patients. It has been well observed by Abercrombie, "In the liver diseases of this country, mercury is often used in an indiscriminate manner, and with very undefined notions as to a certain specific influence, which it is supposed to exert over all the morbid conditions of this organ. If the liver be supposed to be in a state of torpor, mercury is given to excite it; if in a state of acute inflammation, mercury is given to moderate the inflammation and reduce its action.'

"This indiscriminate use of mercury has resulted from its unquestionable efficacy in some derangements of the liver, and from the difficulty of distinguishing the different disorders of this organ. In doubt as to the real nature of the malady, the practitioner is naturally anxious to give his patient the chance of a remedy that occasionally produces marked benefit; but often, in doing so, aggravates the disorder it is his object to relieve.

"This misapplication of mercury will continue until the various diseases and derangements of the liver are better discriminated, and practitioners have ascertained those in which mercury has a curative influence. There can be no doubt, that much of our uncertainty as to the action of this and other medicines, arises from our confounding under the same name, and treating in the same manner, diseases that spring from different causes, and are essentially different in their nature.

"It seems to me that mercury is peculiarly unsuited to the disease we have been considering—suppurative inflammation of the liver.

"One objection to its employment in this disease, is the short time allowed for its action. When the inflammation is consequent on a wound or injury, and also, in all probability, when it occurs in the course of dysentery, it passes on to suppuration in two or three days; and when suppuration has once taken place, and abscess has formed, it is agreed by all who have had experience on the subject, not only that mercury does no good, but that in whatever quantity it be given, it fails to produce its usual constitutional effects. This fact, singular as it may appear, seems to be fully established. Annesley says, 'There can be no doubt that the system will not be brought under the full operation of mercury, or that ptyalism will not follow on the most energetic employment of this substance, where abscess exists.'

"He repeats this opinion again and again, and even considered resistance to the action of mercury, a proof that abscess had formed in the liver.

"It is only, then, before suppuration has taken place, that mercury can do any good, and during this time, from the presence of high fever, the system is with difficulty affected by it.

"When abscesses have formed and become encysted, the time for active treatment by medicine has of course passed away. The wisest course, then, is, I believe, merely to regulate the bowels by rhubarb, or rhubarb and aloes, to recommend habits of strict temperance, and, where the circumstances of the patient allow, residence in a mild climate. If the complexion be sallow or dusky, the nitro-muratic acid, as recommended by practitioners in India, will often be productive of benefit. Whenever there is reason to infer, from increase of pain and fever, that fresh inflammation is set up within the cyst, and that the abscess is growing larger, blood should be taken from the side by leeches or cupping, or a blister should be applied there.

"Many physicians have recommended that abscesses of the liver should be opened; but there is much danger in the practice."

Gangrenous inflammation of the liver is next treated of.

This disease is not common, but when cases do occur, they are found generally, if not always, to be produced by morbid matter transmitted to the organ in the blood from other parts affected with gangrene.

In support of this conclusion several cases are related, one in which abscesses in the liver and lungs followed a contused wound of the finger; another in which mortification of the toes from cold, caused rigors and typhoid symptoms, death on the sixth day, gangrene of the liver, the lungs and spleen, ulceration of the pharynx, and a deposition of pus in the shoulder joints.

"In this case the existence of gangrene, both in the liver and in the lung, was clearly shown by the defined line surrounding the gangrenous portions.

"The source of the mischief here was, no doubt, the gangrene of the toes produced by cold. The man was in the prime of life, of spare habit, muscular, florid, and in good health at the time of the frost bite. The case shows us what a serious thing a small patch of gangrene, in any part of the body, may become.

"The dissemination of the gangrenous masses—the existence of a number of them *isolated and at a distance from one another*—proves that the septic agency was conveyed by the blood. The noxious matter, thus disseminated, destroyed the vitality of the tissues on which it acted most strongly.

"The chemical theory of these septic changes is now well known. All parts in which they are taking place, have a tendency to affect other parts brought into contact with them, with the same mode of transformation. The case just related —and it is by no means a solitary one,—offers one of the most interesting illustrations of this theory in the whole range of pathology. But, whatever be the explanation adopted, the fact is certain, and it is one of extreme importance, that gangrene of the extremities, or of any part of the surface of the body, produced by cold, by pressure, or in any other way, has a tendency to infect other and remote parts of the body with the same change."

"It is in this way, in effect of gangrene of some other part, that true gangrene of the liver is most frequently produced. Rokitansky states, that he has several times observed gangrene of the liver, *in connexion with gangrene of the lung;* and has never found it without gangrene of some other part."

*Adhesive inflammation* of the capsule, and of the substance of the liver, and *cirrhosis* are the consequences of inflammation which causes an effusion of coagulable lymph.

"When lymph is effused in greater quantity on the surface of the liver, it causes adhesion of greater extent; and if any of the lymph fall down among the intestines, it will glue adjacent folds of the intestine together.

"When abscess excites adhesive inflammation of the *substance* of the liver, the lymph can never be diffused in this way. It all remains, where first deposited, immediately around the abscess, and forms a cyst for the matter."

"Deep-seated adhesive inflammation of the liver produces different effects, according to the parts it principally involves. Sometimes the lymph is effused almost exclusively into the areolar tissue in the portal canals of considerable size, and if the person die long after this has occurred, all the considerable branches of the portal vein are found surrounded, in some places to a distance perhaps of half an inch, by new fibrous tissue, which by its contraction has drawn in and puckered the adjacent portions of the liver. The remaining portions of liver may be little, if at all, altered in texture, and may be readily scraped away from these indurated portions. The main branches of the vein are pervious, but many of the small twigs that spring from them are obliterated. The parts which these twigs supplied are atrophied, and the liver proportionally reduced in bulk. Where such portions are near the surface, the capsule is somewhat drawn in and puckered. Together with these changes, there are usually, if not always, thick false membranes on the capsule of the liver, or extensive adhesions, by means of old tissue, between the liver and adjacent organs. Usually, too, there are old false membranes on the surface of the spleen, and marks of adhesive inflammation of other parts, especially the pericardium and the pleura.

"I have several times met with this form of disease in persons who had drunk hard of spirits. It comes on with well-marked symptoms of inflammation of the liver,—pain in the side, vomiting, fever, and perhaps jaundice. These symptoms subside after a time, but the patient does not regain his former health. The liver has been permanently damaged; part of its secreting substance becomes atrophied from closure of the small portal veins, and it is no longer adequate to its office. The patient has difficult digestion, looks sallow, and does not recover his former strength.

"In other cases of deep-seated adhesive inflammation of the liver, the lymph is not effused solely, or chiefly, in the large portal canals. We do not find the fibrous tissue about the large branches of the portal vein especially, but about the small twigs that separate the lobules. All the substance of the liver is rendered tough by this new fibrous tissue, which, when the liver is sliced, is seen to form thin lines between small irregular masses of lobules. At the parts on the surface of the liver which correspond to these lines, the capsule is drawn in, so that the surface has a 'hob-nailed' appearance.

"The tissue of the liver is paler than natural, from the presence of this white fibrous tissue, and from its containing but a small quantity of blood; and it is often yellowish from accumulation of biliary matter in the cells. When this is the case, a section has the grayish and yellow colour of impure bees-wax, and, in consequence, the disease has been called by the French, *cirrhosis*."

"*Causes.* There are perhaps various conditions capable of producing, or that may help to produce, the different forms of adhesive inflammation of the substance of the liver under consideration, but the most common and most powerful cause in this country, indeed the only cause whose influence is apparent, is spirit-drinking."

"The inflammation of the areolar tissue in the portal canals is probably owing to the diffusion of alcohol through it from the portal veins. We can readily fancy such diffusion taking place, if we consider how volatile alcohol is, and how readily it permeates animal membranes and tissues. These properties of alcohol also explain the circumstances noticed by most pathologists, that in cirrhosis the *whole* liver is changed in structure, and the different parts of it generally in pretty equal degree.

"If globules of mercury or of pus find their way into the veins that feed the vena portæ, they become arrested at particular *points* in the lobules of the liver, and excite at each of those points circumscribed inflammation and abscess, while the rest of the liver may continue healthy; but alcohol, being volatile, and mixing readily with water, becomes equally diffused through the whole mass of portal blood, flowing through the liver, and the inflammation it excites involves in consequence the entire organ."

"*Symptoms.*—Cirrhosis usually comes on very insidiously, and when the inflammation does not involve the capsule of the liver, the symptoms are in most cases few and obscure, until the fibrine effused in the substance of the liver has caused impediment to the flow of the portal blood, and to the secretion and escape of bile. Some enlargement of the liver, a dull pain in the right hypochondrium, and disordered digestion, are the chief symptoms in the early stages, and some of these even may be wanting, or be so slight as to escape our notice.

"In some cases however, the onset of the disease is more sudden, and the symptoms at first are more striking and more indicative of active inflammation. The patient has fever, with loss of appetite, perhaps occasional vomiting, and, it may be, jaundice, and his urine is high-colored and charged with lithates. There is much pain and tenderness in the region of the liver, and the liver is readily felt to be enlarged.

"The disease begins in this way when the lymph is effused at once, and the inflammation involves the capsule of the liver.

"When the acute symptoms are subdued by treatment, or subside of themselves, the patient follows his usual occupations, and presents only the slight tokens of disease before mentioned. But he finds that he gradually grows weaker and thinner, his appetite is uncertain, his skin becomes dry and rough, and his complexion sallow and earthy.

"After the lapse of some weeks, or months, or years,—according to the quantity of lymph first effused, the success of the treatment then adopted, and the subsequent habits of the patient—the fibrine poured out has become so contracted, and is in such quantity, that the free passage of the blood through the liver, and perhaps also the free escape of bile from it, is prevented. There then occur a different train of symptoms, which are so characteristic, that there is little difficulty in detecting the disease.

"The belly becomes enlarged from effusion of serous fluid into the cavity of the peritoneum, which takes place without pain or tenderness, and gradually increases so as to cause great distension of the belly, and often, by impeding the movements of the diaphragm, much difficulty of breathing. In some cases this dropsy of the belly is followed by œdema of the legs, but there is no œdema of the hands or face, unless there be likewise disease of the heart or kidneys.

"The patient is liable to hemorrhage from the bowels, and to piles, and the veins on the surface of the belly are enlarged. This enlargement of the cutaneous veins shows clearly that the current of the portal blood is impeded, and is very characteristic of the disease we are considering.

"The complexion is sallow and earthy, or of a slightly greenish cast, and the skin is almost invariably dry and rough."

"It will readily be seen, that most of the symptoms of the advanced stage of cirrhosis result from obliteration or compression of the small twigs of the portal vein, and the consequent obstacle to the circulation through the liver. The blood in the portal vein cannot pass through the liver with its usual freedom, the veins that go to form the portal vein become, in consequence, distended, and various effects follow."

One of the diseases most commonly produced by cirrhosis of the liver is *ascites*. In consequence of the obstruction to the passage of blood from the portal veins, the serum transudes through the coats of the vessels, filling the abdominal cavity and producing all the distressing symptoms consequent upon dropsical effusion into the peritonial cavity.

Another effect of this condition of the liver, is a congested condition of the vessels of the intestines, giving rise to piles and not unfrequently to discharge of blood from the stomach or bowels.

The blood impeded in its passage through the liver, finds its way to the heart, through the cutaneous veins which are in consequence distended.

"*Treatment.*—From what has been already said of the nature of cirrhosis, it is quite clear, that it is only in the early stage of the disease that we can materially benefit the patient. During this stage, while the inflammation is active, it may perhaps be in our power to lessen the amount of effusion, and before the lymph effused has become organized, even to cause its removal by absorption. But when fibrine has been thrown out in large quantity, and when it has become organized, or is otherwise incapable of removal, and has already by its contraction caused much impediment to the flow of portal blood, and materially impeded the due secretion of bile, medical treatment can be only palliative. It is, therefore, of the utmost importance that the disease be detected early, in order that we may be able to obviate such grave and irremediable effects. But, as we have seen, this is not without difficulty, as the symptoms are then often few and very obscure, and it is only by considering the previous habits of the patient, that we see in them the early tokens of organic disease. In the person of a spirit-drinker, we should never neglect pain and tenderness in the region of the liver, especially it associated with some degree of fever.

"At the commencement of the disease, the best treatment is, cupping over the liver, with saline medicines and low diet. While there is much tenderness, and the patient is feverish, nothing produces so much relief as cupping. We must bear in mind, however, that hard drinkers bear bleeding ill, and be careful not to push this remedy too far. Delirium tremens, or other alarming disorder, may be the consequence of its rash and inordinate employment. When bleeding is not considered safe, much benefit may be derived from the application of a blister.

"When the fever has abated, and the liver is still large, mercury and iodide of potassium are the medicines from which most benefit may be expected. Blue pill may be given in moderate doses, so as slightly to affect the mouth; or iodide of potassium may be given internally, and, at the same time, the iodine ointment be rubbed into the side."

Inflammation of the veins of the liver, like phlebitis in other parts, may be suppurative, that is, it may lead to the formation of abscess, or it may be adhesive and lead only to the effusion of coagulable lymph which blocks up and obliterates the vein.

Inflammation of the trunk of the vena portæ is not common for the reason that it is deep seated, and consequently less liable to wounds and other injuries. Cases are related, however, in which inflammation and abscess of the liver were consequent upon suppurative inflammation of this vein.

"Pus-globules brought to the liver by the portal vein, usually become all arrested there, and do not pass through, as they often do through the lungs, to cause scattered abscesses in other organs. It is for this reason that suppurative inflammation of a vein that feeds the vena portæ, kills less quickly than suppurative inflammation of a vein that returns its blood immediately to the lungs. The blood is filtered, as it were, of pus, in passing through the liver, and the local disease is confined to that one organ."

"Mere adhesive inflammation of branches of the portal vein, does not prove fatal, like suppurative inflammation; and on this account, and from the difficulty of distinguishing the different inflammatory diseases of the liver during life, we cannot yet give its clinical history. The patient recovers, and when he dies, perhaps some years after, of another disease, we see merely the ultimate changes to which obliteration of branches of the portal vein leads. These changes are very striking and characteristic. The surface of the liver is marked by deep linear fissures, corresponding to the obliterated branches of the vein, and caused by atrophy of those portions of the liver which the obliterated branches supplied."

The subject next treated of, is inflammation of the gall-bladder and ducts.

"Inflammation of the gall-bladder and ducts probably arises from various causes, each of which determines in great measure the character and the course of the inflammation, and its mode of termination—so that we cannot expect a satisfactory account of the different kinds of inflammation until we can arrange them according to the causes by which they are respectively produced."

"The different forms of inflammation of a mucous membrane, considered with reference to their effects, are

"1st. What may be called *catarrhal* inflammation, which merely increases the quantity and changes the quality of the natural *mucus*, often rendering it viscid, whitish, and opaque."

"2d. *Suppurative* inflammation, where the matter secreted is purulent.

"3d. *Croupal* or *plastic* inflammation, where the matter effused forms a solid albuminous layer on the diseased surface, of which, when this is a tube, it becomes a perfect *cast.*

"4th. *Ulcerative* inflammation—if, indeed, the process which leads to ulceration can with propriety be classed with those leading to the results before mentioned, and be comprehended with them under the generic term, *inflammation.*"

"Catarrhal inflammation of the ducts is, probably, not uncommon. It is not a fatal disease, and, like catarrhal inflammation of other mucous membranes, may cause no permanent changes; so that it may often have occurred, where no traces of it are found. It happens, however, not very unfrequently, that on squeezing the hepatic ducts, a viscid whitish fluid oozes out, which, on examination through the microscope, is seen to be chiefly made up of the prismatic epithelial cells of the gall-ducts. The symptoms we should expect in catarrhal inflammation of the hepatic ducts, are some degree of feverishness, with slight pain in the region of the liver, and it many of the ducts become closed by thickening of their coats, or be choked by the viscid secretion, slight enlargement of the liver, and jaundice.

"Many of the cases of simple jaundice coming on in healthy persons, and attended with very little pain and fever, are probably cases of this kind.

"In a severer form of inflammation, the matter secreted is purulent, but it has seldom the visible characters of pure pus. The pus is mixed with opaque mucus secreted at the same time, and, it may be, with bile also. If the bile be in considerable quantity, and ammoniacal, its alkali renders the pus glairy, and the result is a viscid, greenish, or yellowish fluid, very different in appearance from pure pus."

"Suppurative inflammation of the gall-bladder seems especially liable to occur when, by any cause, the cystic duct is permanently closed.

"Cruveilhier (liv. xxiii. pl. 5) has given a plate of a liver studded with cancerous tumours, in which the cystic duct was obliterated, and the gall-bladder inflamed and full of pus. No notes of the case are given.

"A similar instance is recorded by Andral, (Clin. Med. iv. 518,) in the case of a woman who died at the age of 47. There were numerous cancerous tumours in the liver. The gall-bladder was full of pus, and its mucous membrane inflamed. The cystic duct seems to have been closed. The hepatic duct was very large and full of bile. The common duct exhibited nothing unusual. There was recently effused lymph on the surface of the peritoneum, and the mucous membrane in the large end of the stomach was softened. No other marks of disease are noticed.

"Inflammation of the gall-bladder, whether catarrhal or suppurative, seldom, perhaps, proves fatal of itself, except when the cystic duct is closed, and the gall-bladder converted into an abscess. When it is the sole disease, and the ducts are open, so that the matter can escape, the patient may, perhaps, recover perfectly, or may survive with the gall-bladder more or less changed in structure. I have twice found the gall-bladder and cystic duct contracted, and their coats thickened, in young persons who died of other diseases, and in whom there were no gall-stones, nor any trace of inflammation of the common or hepatic ducts, or of the capsule or substance of the liver."

"*Croupal* or *plastic* inflammation of the mucous membrane of the gall-bladder and ducts is very rare. Rokitansky says he has observed it in the ducts within the liver, in what has been called the secondary fever of cholera, and as a sequel of ordinary typhoid fever. It produces within the gall-ducts membranous tubes, in which the bile forms tree-like concretions; and this again, by blocking up the passage, causes distension of the capillary ducts behind."

"*Ulceration* of the gall-bladder is much more common than the forms of inflammation yet considered, and occurs in various circumstances."

"Sir G. Blane, in his account of the Walcheren fever, states that the mucous membrane of the gall bladder was frequently found inflamed and ulcerated; the ulcers having in some cases the conical or tubercular form sometimes seen in dysentery. The gall bladder was generally distended with bile, which, in those persons who died early, was of a deep green or dark brown, but in more protracted cases had the consistence and colour of tar. This tar-like fluid did not taste bitter like bile, and when mixed with water did not impart any yellowness to it, while it was often so acrid as to excoriate the lip."

"The acrid quality of the bile in the Walcheren fever, and the circumstance that in Dr. Boyle's dissections, the strongest marks of inflammation in the intestinal canal were about the entrance of the common duct into the duodenum, render it probable that the inflammation of the gall-bladder and duodenum, in remittent fever, is caused by irritating bile. As in typhoid fever, the symptoms of inflammation of the gall-bladder are not distinguishable in the midst of the general disorder that constitutes the fever, and the symptoms of inflammation of other parts that likewise occur in its course.

"In this country, ulceration of the gall-bladder is produced perhaps not unfrequently by the irritation of gall-stones.

"Ulceration of the gall-bladder and gall stones are often found together, but we must not infer in all such cases, that the ulcers were produced by the gall-stones. Both the ulcers and the gall-stones may have resulted from the presence of bile of unnatural quality.

"When there is only one ulcer in the bladder, and a large or hard gall-stone is found resting upon it, we may perhaps safely infer that the mechanical irritation of the gall-stone was the cause of the ulcer. Gall-stones too large to pass through the cystic duct, not unfrequently cause ulceration of the lower or depending part of the gall-bladder; lymph is poured out on the peritoneal coat below the ulcer; the gall-bladder becomes united by this means to the duodenum or colon; the ulcer eats likewise through the coats of the intestine, at this point; and the gall-stone escapes into the intestinal canal. The processes of ulceration and adhesion take place very slowly,—and are seldom attended by alarming symptoms. Often, indeed, the first clear intimation that such an event has happened, is the discharge of a large gall-stone from the bowels.

"In other cases, we find many small round ulcers in the gall-bladder, and perhaps in the common duct, and small gall-stones in the bladder not resting on the ulcers. When it is considered that most human gall-stones are so light as to float in bile—since they almost float in water, which is of much lower specific gravity—and that, consequently, they can exert no pressure on the coats of the gall-bladder from their *weight*, when there is bile enough in the bladder to keep them afloat;—it seems most reasonable to refer both ulcers and gall-stones in these to an unhealthy state of the bile."

"Ulceration of the gall-bladder and ducts may lead to various results.

"1st. An ulcer, commencing in the mucous membrane of the gall-bladder or of the common duct, may eat through its different coats until the peritoneal coat is laid bare. The bile, brought in contact with this coat, causes it to slough, and the contents of the gall-bladder are poured suddenly into the cavity of the peritoneum. When this happens, diffuse suppurative inflammation of the peritoneum is set up, which destroys life in a few hours—quicker, perhaps, in most cases, than the peritonitis that follows rupture of the bowels."

"2d. When an ulcer of the bladder or ducts is caused by a gall-stone, adhesive inflammation of the serous membrane is usually set up before perforation takes place; the gall-bladder or duct becomes united to some adjacent part, generally the duodenum or colon; the coats of the intestine are eaten through after those of the gall-bladder or duct; and the gall-stone passes into the intestinal canal."

"3d. Ulceration of the gall-bladder or ducts, like ulceration of other mucous surfaces that return their blood to the portal vein, may lead to scattered abscesses in the substance of the liver."

"The abscesses are probably the immediate consequence of suppurative inflammation of a small vein in the vicinity of the ulcer, or of the absorption of the ichorous matter of the ulcer."

"Closure of the *cystic* duct destroys the office of the gall-bladder, and leads to various changes in it, which depend chiefly on the length of time the duct has been closed, and on the previous condition of the gall-bladder.

"When the cystic duct is closed by adhesive inflammation of the capsule of the liver, and the coats of the gall-bladder were previously healthy, the bile in the gall-bladder is absorbed, and its place is soon occupied by a glairy fluid, of the consistence of mucus or synovia, and not at all tinged, or but very slightly tinged with bile. After a time, this fluid is secreted in less abundance, and the gall-bladder contracts and shrivels; in some cases, almost to the size of an almond."

"The effects of closure of the cystic duct on digestion and the general health, are, much less serious than might have been expected, and sometimes are of very little import."

"Closure of the *common* duct has far more serious effects.

"The most immediate of these, are deep jaundice, dilatation of the gall-bladder and hepatic ducts, and retention of bile in the lobular substance of the liver, which acquires in consequence a deep olive colour. By the retention of bile, the liver at first grows larger, but its increase of size from this cause is, perhaps, never very great. Subsequently, from atrophy of the lobular substance, it shrinks again, and, in the end, notwithstanding the dilatation of the gall-ducts, becomes much smaller than in health.

"If the closure of the common duct occur suddenly, the gall-bladder, or one of the ducts behind the obstruction, may be distended so rapidly as to burst. Several cases of this kind are recorded."

"The ultimate effect of closure of the common duct on the *lobular substance* of the liver, is very remarkable. The cells which go to form this substance, and which secrete the bile, are destroyed; the capillary vessels of the lobules, which minister to secretion, their office gone, waste; the liver shrinks and no longer presents an appearance of lobules, and its office is no longer in any degree performed."

"In the *treatment* of inflammation of the gall-bladder and ducts, a most important principle is the early employment of local depletion. Leeches, as was seen distinctly enough in some of the cases that have been related, relieve the pain and tenderness, and no doubt mitigate the inflammation, and, in consequence, lessen the danger of perforation and of permanent closure of the ducts."

"Blisters have the same kind of efficacy as leeches. Like these, they often relieve the pain and tenderness in a striking manner, and therefore, we may infer, tend also to prevent permanent changes of structure. The proper time for blistering is when the pain and fever have abated under leeches and other measures, and it is no longer deemed advisable to take away blood.

"Another important principle in the treatment of these cases, is the strict enforcement of a plain and appropriate diet. As a particular point in the diet to be observed, the free use of diluents may have some advantages. While, by filling the stomach, they help to empty the gall-bladder by their pressure, it is also probable that, after absorption, they pass out of the circulation again, in part by the liver, and thus dilute the bile.

"In certain cases of the class now under consideration, the judicious use of mercury is attended with signal good effects. It probably acts beneficially in two ways:—1st, by increasing the quantity and by promoting the flow of bile; and, 2d, by producing changes in its quality which render it less irritating. These are the objects that determine the principles of its administration in these cases, in which the desired effect is best obtained, not by the more powerful and constitutional action of the drug,—which should be studiously avoided,—but by the occasional administration of its milder preparations, repeated as need may be. It is to the striking benefit sometimes derived from mercury used in this way, that this medicine owes the reputation it has long had as a remedy in liver diseases.

"Soda is another medicine much in use in the treatment of these cases, and there is reason to believe that it deserves the esteem in which it is generally held. Physiological considerations would lead us to suppose that it is best suited to cases of catarrhal inflammation of the ducts. As soda is a natural constituent of bile, and is therefore,—we may infer,—readily excreted by the liver, it probably renders the secretion from the ducts less viscid, and has the same sort of efficacy in these cases as in catarrhal diseases of the lungs, in which this and other alkalies have been long used as expectorants."

"Having considered the inflammatory diseases of the liver," the author next passes to the consideration of "diseases, in which, seemingly without inflammation, the secreting power or the nutrition of the heptic cells and other tissues of the liver, is seriously disorded.

"These diseases may be divided into two principal groups. One of these groups is characterised by suspension of the secretion of bile. The principal feature of the other is, that the hepatic cells separate from the blood, some abnormal matter, which, instead of passing freely out of the liver in the bile, is retained there, adding to the size of the liver, and more or less changing its appearance and texture."

"The most remarkable and most serious change is, where the cells are completely broken down and destroyed. It has been seen that this may result from long retention of the secreted bile from closure of the common duct. In consequence of this the hepatic gall-ducts become enormously dilated, and the whole liver acquires a deep olive colour. Its tissue is flabby, but not readily broken down by the finger, and *presents no appearance of lobules.* Every part of the liver is affected alike, and exhibits under the microscope nothing but free oil-globules and irregular particles of solid biliary matter. The liver contains but little blood, and partly from this, but chiefly from loss of the cells, it may be smaller than in health, and its surface wrinkled, notwithstanding the biliary matter accumulated in it.

"But destruction of the hepatic cells may take place rapidly without any obstruction of the gall-ducts and instead of being consequent on jaundice, may be the cause of jaundice that proves rapidly fatal, apparently from disorder of the functions of the brain.

"It has been long known that cases of jaundice now and then occur which prove fatal in this way; and that in such cases it frequently happens that no obstruction can be found in the gall-ducts,—which are pale and empty of bile,—and no effusions characteristic of inflammation in any part of the liver. In some such cases, no change of structure has been remarked in the liver, and the disease has been described as fatal jaundice from suppressed secretion. In other cases, the liver has been found unusually small, and much softened, and changed in colour, and the disease has been spoken of as *softening* of the liver, or *simple softening* or *black softening*, according to the colour of the liver in the individual case."

"It would seem that this suspension of the secreting process and disorganization of the liver, may result from powerful and depressing emotions; but that it is far more frequently produced by some poison, introduced from without, or generated in the body by faulty assimilation or digestion. It appears, too, that various poisons,—pus, the poison of serpents, perhaps the poison of some forms of fever, and various others,—may alike stop the secretion of the liver, and lead to the same kind of disorganization of its structure, while their other effects on the system are very different."

It has already been stated, that the size, color and firmness of the liver may be changed without the intervention of inflammation, and without any destruction of its cells, simply from matter being secreted in undue quantity, which, instead of passing off freely in the bile, is retained in the substance of the organ.

The most common disease of this class is fatty liver, or fatty degeneration of the liver.

In every human liver, there are always deposited in the secreting cells, small quantities of oil or fat.

"In the fatty liver, the quantity of oil so placed is enormously increased. The hepatic cells are gorged with large globules, which greatly distend them, and often obscure their nuclei."

"A liver that has undergone the fatty degeneration, may be little altered in shape, but it it is larger, and paler, and softer, and more greasy than natural. These changes in its sensible qualities depend chiefly, if not solely, on the interstitial deposit of the oil-globules, and their degree may give us some estimate of the quantity of oil the liver contains. When this is very large, the liver is large in proportion, sometimes twice its natural size, and is generally somewhat altered in shape, being thicker than natural, and having its edges blunter or more rounded. The capsule of the liver is stretched and smooth, and when divided its edges recede. The tissue of the liver is pale, and, generally, throughout of a soft buff colour, dotted with brown or red. The brown or red dots mark the centres of the lobules, which are unusually large and distinct, and are buff-coloured near their margins. The liver is very soft, and greases the hands, or the scalpel, like common fat.

"When the quantity of oil is less, the liver is not so large, nor so pale, nor so soft,—but presents an appearance described as the nutmeg-liver. The liver may not feel greasy, but an unusual quantity of fat may be at once detected by placing a thin slice of the liver on a piece of paper, and exposing it to the action of heat. Some of the oil or fat exudes, and greases the paper. The best way; however, of ascertaining the quantity of fat—at least that which exists in the form of oil-globules —is by examining a small particle of the liver through the microscope. The oil-globules are objects of sight, and from their form and their dark outline, are at once distinguished."

"An accumulation of fat in the hepatic cells, notwithstanding it so changes the appearance and other sensible qualities of the liver, seems not to interfere much with its office. There is no jaundice; no congestion of the veins that feed the vena portæ,—no obstruction, therefore, to the circulation through the liver; no pain, or even tenderness. The only inconvenience the patient suffers from this condition of the liver, is that which arises from the bulk of the organ,—distension of the belly, and a sense of fullness and weight, on turning in bed from the right side to the left. The reason of their being no jaundice is, that the colouring matter of the bile is secreted, and passes off, as usual. The absence of other symptoms seems to depend on the softness of the oil-globules, and the readiness with which they change their form and yield to pressure; on their being deposited gradually and evenly, so as not to cause sudden stretching of the capsule of the liver; and on their having no tendency to excite inflammation of the capsule, or of the veins.

"The liver becomes fatty in very different states of the body.

"1st.—It is often fatty in persons who lead indolent lives, and are at the same time gross feeders—eating largely of fatty substances, and drinking freely of spirits, but more especially of porter and other heavy malt liquors; and is then generally associated with excess of fat under the skin, and in other parts of the body in which fat is usually deposited."

"2d.—But the liver is often found fatty in persons dead of phthisis, who, instead of being loaded with fat, are generally much wasted.

"The frequency with which the liver undergoes this change in phthisis, was, I believe, first pointed out by M. Louis, in his celebrated work on phthisis, published in 1825. M. Louis detected the fatty degeneration by the altered look and feel of the liver, in forty cases of phthisis, out of 120,—or, in one-third of the subjects he examined."

"Fatty degeneration of the liver in such degree as to be at once recognised, is not only frequent in phthisis, but,—setting aside the persons in whom the liver is loaded with fat in common with the areolar tissue and other parts of the body in which fat is liable to be deposited,—is almost peculiar to this disease. Frequently, indeed, in subjects dead of various diseases, an unusual quantity of fat is found in the liver, which is at once discovered by the microscope, and which may be detected by a practised eye, by merely looking at the liver,—but the fatty degeneration is seldom so advanced as to be readily recognised at sight, except in persons dead of phthisis. M. Louis states, that in the course of three years he met with forty-nine instances of fatty liver, and in forty-seven of these, the patients were phthisical.

"In speculating on the cause of this peculiar tendency to accumulation of fat in the liver, in phthisis, it is important to remark, that it does not depend on tuberculous disease of the liver itself. M. Louis states, that there were no tubercles in the liver in any of the cases in which he found it fatty: and that in two cases in which there were tubercles in the liver, the liver was not fatty."

"It has been imagined, that fatty matter accumulates in the liver in phthisis, in consequence merely of the office of the lungs being greatly and gradually interfered with—that hydro-carbonaceous matters, passing off in less quantity than natural through the lungs, are, in consequence, eliminated in larger quantity by the liver. This opinion is rendered very improbable by the circumstance, that in organic diseases of the heart, and in asthma, where the office of the lungs is not unfrequently as much interfered with as in phthisis, the liver does not become fatty. Still stronger refutation of it is afforded by the fact, noticed by Rokitansky, that fatty degeneration of the liver is found in conjunction with tuberculous disease of other organs—the mesentery, the serous membranes, the bones—when there are no tubercles in the lungs.

"These facts show that we must seek the explanation of the fatty degeneration of the liver in phthisis, in some other conditions than mere diminished function of the lungs."

"The opinion was some years ago advanced by the late Baron Larrey, that the fatty condition of the liver in these cases results from solution of the fat previously laid up in the body. He considered this opinion strongly supported by the method then employed in France to make the livers of geese fatty, and of which he gives the following account: 'To procure the large livers of geese, for the making of patties, fatted birds are confined in close cages, and then exposed to a graduated heat, being kept at the same time entirely without food, even without water. They become feverish, the fat undergoes a kind of fusion, and the liver grows enormously large. The liver is considered to be in the desired state, when the animal is *extremely wasted*, and the fever increases.'

"It is quiet clear, that, in this process, the fat which accumulates in the liver, is derived from that previously laid up in the body. It is extremely probable, that the same thing happens in phthisis, and in the other wasting diseases in which fatty degeneration of the liver occurs, in man: that in the process of wasting, the fat stored up in the body is largely taken up by the veins, so that it comes to be in excess in the blood, and is then laid hold of by the hepatic cells, which have a natural affinity for it."

"Our knowledge of the frequency of fatty degeneration of the liver in phthisis enables us often to discover it during the life of the patient. In a woman labouring under phthisis, considerable enlargement of the liver, without jaundice, or ascites, or much pain or tenderness, is evidence enough, especially when the woman has been of temperate habits, that the liver is fatty. But as this condition of the liver causes but little inconvenience in itself, and does not lead to inflammation, or to other secondary mischief, and as the disease with which it is associated is inevitably fatal, it is not an object of treatment.

"When the liver becomes fatty from gross feeding and indolent habits, the excess of fat will, doubtless, disappear from it, as from other parts, on the person adopting an opposite mode of life. If he will rise early, take active exercise, live chiefly on lean meat, with plenty of salt, and drink water—and will abstain from butter, bacon, oil, beer and other fermented drinks, and eat sparingly of sugar and potatoes—he will not only get rid of his fat, but his muscles will be better nourished, and his strength be increased."

A condition analogous to fatty liver, is sometimes met with in persons wasted by scrofulous disease, called by writers scrofulous enlargement of the liver.

"Scrofulous enlargement of the liver, like the enlargement from deposit of fat, comes on without pain of the liver, or even tenderness; a circumstance sufficiently accounted for by the gradual and even manner in which the foreign matter accumulates, and from its having no tendency to cause inflammation of the capsule of the liver, or of the veins."

"In this disease, as in the fatty liver, the secretion of bile —or, at least, of the colouring matters of bile—may go on as usual, and the complexion remain quite clear. But this is, perhaps, not so generally the case as in the fatty liver. The matter deposited in the substance of the liver being firmer, is, probably, more apt to interrupt the secretion, or the flow of the bile, and to render the complexion sallow. Dr. Graves has remarked that in persons with scrofulous enlargement of the liver, the stools are variously coloured with bile—"one part of them will be bilious, another part of them clay-coloured; they will be yellow to-day, and pale to-morrow," (Clinical Medicine, p. 566.) He infers from this, that the office of the liver is performed intermittingly; that the liver secretes bile during a certain period of the digestive process, then stops, and then secretes again."

"The *treatment*, in these cases, should have chief reference to the state of the system—the peculiar cachexy—on which the faulty secretion and large size of the liver depend.

"When the enlargement of the liver is consequent on scrofula, our chief reliance must be on warm clothing, sea-air and bathing, a light nourishing diet, comprising a liberal allowance of animal food and wine, and the preparations of iodine and iron, separate or combined.

"When the health has been broken by the combined effects of syphilis and mercury, warm clothing, a tonic regimen, iodide of potassium, nitric acid, sarsaparilla, and guiacum, are the appropriate remedies.

"In either case, the original malady is faulty assimilation, and, if we can remedy this, we shall probably, in most cases, if not in all, remedy the unnatural condition of the liver, and other secondary ailments.

My own experience leads me to think highly of frictions with iodine ointment, long-continued, in such cases. I have several times seen an enlarged liver reduced to its natural volume by iodide of potassium and frictions with iodine, or, simply by these frictions and saline purgatives. The matter deposited in the liver does not become organized like the fibrine poured out in inflammation, and, if the general health mends, it may, in time, pass off in the bile, or be removed by absorption."

The next subjects investigated are diseases of the liver, such as, excessive and defective secretion of bile, morbid bile, gall stones, cancer of the liver, hydatid tumors, &c.; but as these are diseases less frequent and of less importance than those previously treated of; and as we have already extended our quotations farther than we had intended; we will close with the remark, that in our opinion, this work, from Dr. Budd, is one of the best selections for the library of a western physician.

